# New Optical Methods for Liveness Detection on Fingers

**DOI:** 10.1155/2013/197925

**Published:** 2013-09-18

**Authors:** Martin Drahansky, Michal Dolezel, Jan Vana, Eva Brezinova, Jaegeol Yim, Kyubark Shim

**Affiliations:** ^1^Faculty of Information Technology, Brno University of Technology, Bozetechova 2, 61266 Brno, Czech Republic; ^2^Department of Dermatology and Venereology, Faculty of Medicine, St. Anne's University Hospital, Masaryk University, Kamenice 753/5, 625 00 Brno, Czech Republic; ^3^Department of Computer Engineering, Dongguk University at Gyeongju Gyeongbuk, Sekjang-Dong, Gyeong Ju, Gyeongsangbuk-do 780-714, Republic of Korea; ^4^Department of Statistics and Information Science, Dongguk University at Gyeongju Gyeongbuk, Sekjang-Dong, Gyeong Ju, Gyeongsangbuk-do 780-714, Republic of Korea

## Abstract

This paper is devoted to new optical methods, which are supposed to be used for liveness detection on fingers. First we describe the basics about fake finger use in fingerprint recognition process and the possibilities of liveness detection. Then we continue with introducing three new liveness detection methods, which we developed and tested in the scope of our research activities—the first one is based on measurement of the pulse, the second one on variations of optical characteristics caused by pressure change, and the last one is based on reaction of skin to illumination with different wavelengths. The last part deals with the influence of skin diseases on fingerprint recognition, especially on liveness detection.

## 1. Introduction

A first phase of fingerprint processing is obtaining a digitalized fingerprint [1]. The traditional (dactyloscopic) method uses the ink to get the fingerprint onto a piece of paper. This piece of paper is then scanned using a common (office) scanner, but this method is not interesting for our following description. The modern live fingerprint readers are used—they use different physical effects to acquire the image of a finger.

While the first generation scanners used optical techniques [2], a variety of sensing techniques are used today and almost all of them belong to one of the three families [2]: *optical*, *solid state*, and other (e.g., ultrasound). Solid-state sensors are now gaining great popularity because of their compact size, which facilitates embedding them into laptop computers, cellular phones, smart cards, and so forth.

We can define all fingerprint scanning technologies as separate methods, because they do use special physical effects to obtain the impression of a finger—to the known technologies belong [3–6]: *optical*, *capacitive*, *ultrasound*, *e-field*, *electrooptical*, *pressure sensitive*, *thermal,* and *MEMS* (microelectromechanical systems) [7].

When a legitimate user has already registered his finger in a fingerprint system, there are still several ways how to deceive the system. These ways and methods are well known. Further details can be seen, for example, in [8–10]. In order to deceive the fingerprint system, an attacker may use any of them. In this paper, we will focus only on deceiving the fingerprint system by using an artificial finger (print) fakes.

**Figure 1:**
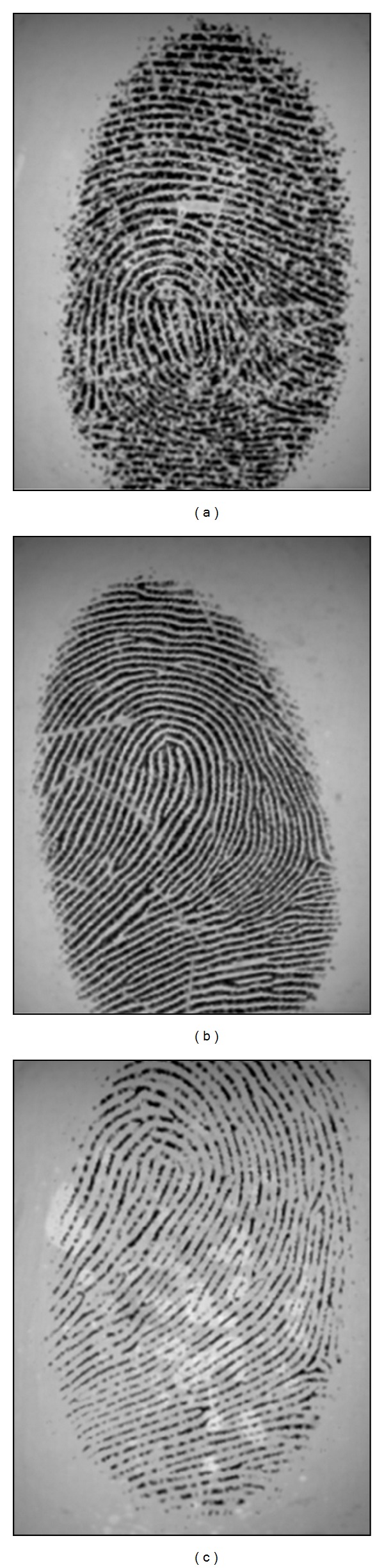
Artificial fingerprints generated by SFinGe [6, 16].

The term *fake samples* (e.g., *fake finger (print)*) [9, 11, 12] may be widely used with reference to biometric samples which are used to deceive biometric systems. However, the term “artificial samples” (e.g., artificial fingerprint) (see Figure 1) corresponds to biometric samples which are entirely artificially produced. Mathematically, “artificial samples” represent a subset of “fake samples”, because the set “fake samples” may also include modifications of live samples [9, 11], for example, “artificial samples” are fingerprints produced from a mold, but the set “fake samples” contains also injured or otherwise modified live fingers and biometric samples.

The traditional authentication used during the enrollment process should be stronger than all subsequent verifications, because each of the subsequent verifications depends on the strength of authentication and the quality of biometric samples acquired during enrollment. However, in many biometric implementations the enrollment process may become a weak link. One possible protection method could be found in [13] or [14] or [15].

The implications of this susceptibility to *spoofing *[19–21]—defeating a biometric system through fake biometric samples—include the following [22].

- Fake finger attacks may be mounted against existing enrollments in order to gain access to a protected facility, computer, or other resource.
- A fake finger may be used for authentication at a given computer or border crossing in order to fraudulently associate an audit trial with an unwitting individual.
- A fake finger may be used to enroll in a biometric system and then be shared across multiple individuals, thereby undermining the entire system.
- An individual may repudiate transactions associated with his account or enrollment—claiming instead that they are the result of attacks—due to the inability of the biometric system to ensure liveness.

Given biometrics' widespread acceptance [22] as a solution for a range of public and private sector applications such as civil identification, network security, border control, and point of sale authentication, the question of liveness detection in leading biometric technologies must be addressed.

The concept of liveness detection can be framed by considering the detection of liveness versus the detection of nonliveness [22]. The liveness detection may take place at the acquisition stage, such that nonlive data are not acquired, or at the processing stage, such that nonlive data are not processed. 

Let us assume that the enrollment process was properly performed and the user has registered his biometric sample, without which it is pointless to assure the system in the verification/identification phase. In general, three basic attacks are feasible whenever the liveness detection is not well implemented [11, 23].

- *Presenting artificial samples of the registered user* is the most common and most important threat or risk in this category because of the relative easy effort and variety of ways in which such an attack could be realized. Artificial samples could be produced in many ways from many materials, which poses a problem for detection countermeasures. Dishonest acts with artificial samples could be divided into two classes: artificial samples produced with the assistance of the registered user and artificial samples produced without his assistance.
- *A latent sample reactivation* risk relates to touch fingerprint systems. The rarely used sweep sensors do not have to address this problem, because the method for imaging includes a self-cleaning function during each capture. In addition to the liveness detection, touch fingerprint systems should include a cleaning mechanism after each imaging. It is worth noting that a biometric system should reveal under no circumstances the information (like a score or threshold) to the user that could be useful for attackers. Additionally, before initiating another transaction, a biometric system should clear all biometric data from the memory, to ensure its security in a case when the attacker gains control of the system.
- Last but not least, there is a *severed sample*—as mentioned before.

To overcome the above mentioned threats or risks, the *liveness detection* [24] should be implemented. In an environment where a higher level of security is required, a biometric system should be a part of two- or three-factor authentication solution. There are other attacks at the sensor level but they are possible whether or not liveness quality is implemented.

The liveness testing may take place at the acquisition stage, when nonlive data are not acquired, or at the processing stage, when nonlive data are not processed.

Another very important feature for the security and proper working of a biometric system is to assure that the capture of the biometric sample and measurement of liveness occur at the same point in space and time [11]. Otherwise, an attacker may present his live biometrics to pass the liveness testing and then he may deceive the verification process by supplying an artificial sample.

It is clear not only from [4, 9] that the production of a fake finger (print) is very simple. Our own experiments have shown that to acquire some images (e.g., from glass, CD, film, or even paper) is not very difficult and, in addition, such image could be enhanced and postprocessed, which leads to a high-quality fingerprint. The following production process of a fake finger (print) is simple and can be accomplished in several hours. After that, it is possible to claim the identity as an impostor user and common (nearly all) fingerprint recognition systems confirm this false identity supported by such fake finger. Therefore, the application of *liveness detection* methods is a very important task and should be implemented (not only) in all systems with higher security requirements, such as border passport control systems and bank systems. 

## 2. New Optical Methods for Liveness Detection

In the following subsection, three new principles of liveness detection will be described, which are based on optical changes— the first is based on fine movements of fingertip area measured by a laser triangulation module (Section 2.1); the second one is based on measurement of optical characteristics after pressure change on a finger (Section 2.2); the last one is based on measurement of optical changes based on illumination of a finger by light with various wavelengths (Section 2.3).

### 2.1. Measurement of Pulse Based on Optical Measurement

The interaction of light with matter (here with human tissue) can be basically described in the terms of absorption, scattering, and fluorescence. The light from the illumination like LED, laser, or other sources hits the skin and is partially scattered on the surface and partially enters the tissue in which it will be absorbed, scattered, or reemitted. So when illuminating the finger from the side a fraction of scattered and reemitted light leaves the finger in approximately 4*π* direction and can be detected in other direction. Such scattered light may include information about dynamical processes like blood flow, hemoglobin saturation, and pulse from inside the finger. Scanners based on this technique try to detect whether the scanned object exhibits characteristics of the pulse and blood flow consistent with a live human being [11]. It is not very difficult to determine whether the object indicates some kind of pulse and blood flow, but it is very difficult to decide if the acquired characteristics are coincident with a live sample. As a result, it is difficult to create an acceptance range of the sensor, which would lead to small error rates. The main problem is that the pulse of a human user varies from person to person—it depends on the emotional state of the person and also on the physical activities performed before the scanning procedure. In addition, the pulse and blood flow of the attacker's finger may be detected and accepted when a wafer-thin artificial sample is used.

Depending on the composition of the original light spectrum, the illuminated object appears in colors which result mainly from the absorption spectrum of the material secondly and much less from the angle of scattering and partially from material capability for fluorescence itself.

One example of an optical skin property is the scattering on skin surface and the absorption in the tissue mentioned above—the light illuminating the surface is partly scattered and partly absorbed and reemitted. The light detector acquires the outgoing light spectrum which has been changed in intensity due to absorption and eventually due to the fluorescence of the proteins in the tissue. Another example for optical skin feature is the saturation of hemoglobin [14, 17, 25], which binds oxygen molecules. When blood comes from the heart, oxygen molecules are bound to the hemoglobin (oxyhemoglobin), and, vice versa, when blood is flowing back to the heart, it is less saturated by oxygen (deoxyhemoglobin). The absorption properties of these two hemoglobin molecules are different that means the color of oxygenated blood is different from that of nonoxygenated blood. If we use a light source to illuminate the finger tissue, we can follow the blood flow based on the detection of oxygenated and nonoxygenated blood, respectively [17]. 

**Figure 2:**
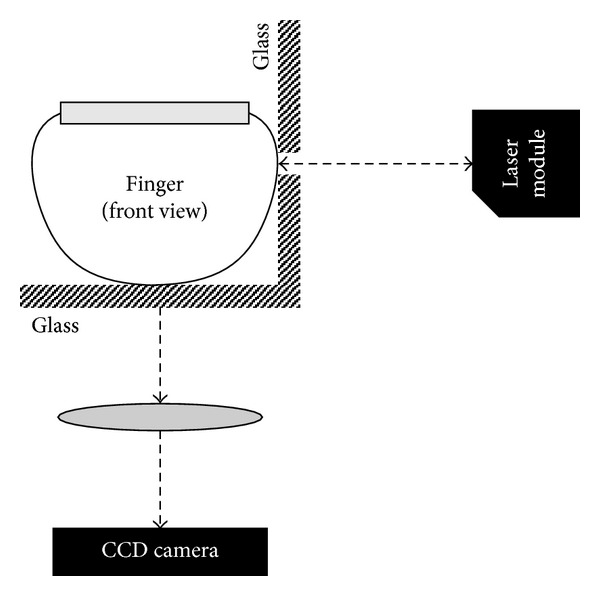
Possible integration of laser distance measurement for liveness detection with optical fingerprint sensor [17].

Another solution is proposed in [17] based on the analysis of movements of papillary lines of the fingertips with the help of the so-called high precise laser distance measurement technique. One advantage of this implementation is that the finger is not required to be in contact with a specific measuring device, and so it can be integrated with standard fingerprint sensors, Figure 2.

The laser distance measurement [17, 26] module is placed to the right side of the glass plate, which is L-shaped. The user places his finger in such a way that it is in contact with the horizontal and the vertical side of the glass plate.

The underlying physical measurement principle is as follows. A semiconductor laser is used to produce a laser beam which illuminates the finger. Because of the scattering of the coherent laser wave fronts on the fingertip skin, a part of the laser spot is reflected back to sensor (position sensing device). This light interferes there and produces characteristic speckle pattern whose dynamic change is used for very precise triangulation measurement. Although the method is not suitable for resolving the papillary lines itself, it measures the small dynamic fluctuations of the papillary lines due to the heart beat and muscle tonus.

The comparison of the computed curve and a normalized standard curve (the template) will reveal whether the measurement corresponds to a standard live fingerprint or indicates a fake finger or another attempt of fraud. For example, the comparison between both curves can be realized by the normalization followed by the cross-correlation.

The optical bench consists from the following parts:

- Panasonic LM10 microlaser displacement sensor,
- Panasonic LM10 sensor single comparator,
- special holder for Panasonic LM10 sensor,
- real-time digital phosphor oscilloscope Tektronix DPO7254.

**Figure 3:**
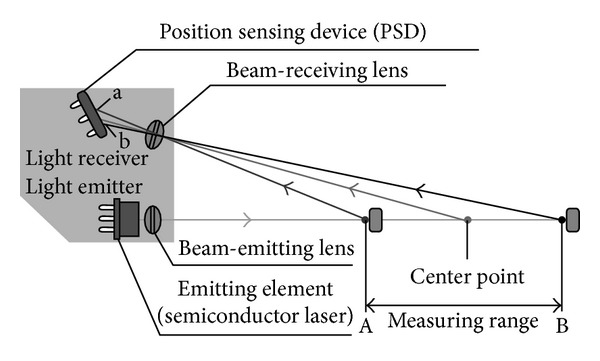
Measurement principle of the Panasonic LM10 microlaser displacement sensor [18].

A principle of the distance measurement using LM10 can be found on Figure 3.

The optical bench uses the Panasonic LM10 microlaser displacement sensor for measuring the distance between the finger and the sensor head. The measurement principle of LM10 is based on optical triangulation. A semiconductor laser is used to produce a laser beam which illuminates the finger. Because of the diffuse reflection of the laser beam on the fingertip skin, a part of laser rays is reflected back to sensor. A light spot from the reflected laser rays is tracked by a sensor part called position sensing device. The position sensing device measures the distance between fingerprint and sensor measuring the fluctuations of a light spot position. A diagram of the distance measurement process [27] can be seen in Figure 3.

**Figure 4:**
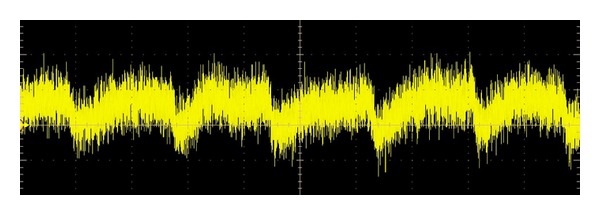
Result of liveness detection—volunteer 1.

**Figure 5:**
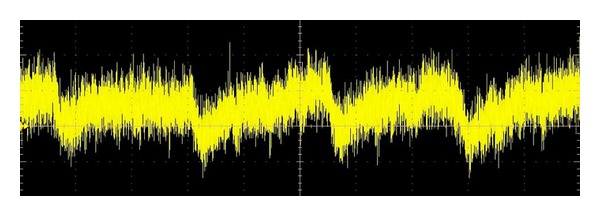
Result of liveness detection—volunteer 2.

The curve with heart activity (pulse) visible from the measurement using this method based on laser measurement principle can be seen in Figures 4 and 5.

In Figures 4 and 5 you can see the liveness detection results of two randomly chosen volunteers. Volunteer 1 is a woman, age 25. Volunteer 2 is a man, age 27. Both volunteers were before and during the experiments calm, rested, and in good physical and psychical condition. Results of this measurement are the curves with clearly visible heart activity (similar to classic electrocardiogram curve). As a matter of interest, it can be seen that the volunteer one (woman) has a faster pulse during the experiments.

There are other liveness detection methods based on optical principles—see the following sections.

### 2.2. Measurement of Optical Characteristics in the Finger Based on Pressure Change

In our team, a novel approach was proposed based on combination of detection of two characteristics of live human fingers (change of color and elasticity due to pressing of finger against glass plate)—closer information with testing results could be found in [28] and this section is based on [28].

Under normal circumstances, a live human finger is reddish and its papillary lines are approximately 0.2–0.5 mm wide (The width of papillary lines differs from one person to another, but it depends on various conditions, e.g., age of the person.). Due to the pressing of finger against glass plate, the height of papillary lines decreases so that the lines optically appear to be thicker and the blood is partly relocated from the pressed skin area so that the skin turns to yellowish/whitish [28, 29]. Once the pressure on the finger is decreased (or eliminated), the papillary line color and optical thickness immediately come closer (returns back) to its original state. However, the percentage of extension of width of papillary lines and its color are not always the same. The rate of change is proportional to the force of finger pressing.

The color of finger (and also the color change) can be detected using various color models. The experiments with various color models could be found in [28], for example, RGB, HLS, or CIE L*a*b*. The results of experiments with HLS color model shows that this model is not convenient for purposes of this liveness detection due to the high intraclass variability.

The results of tests in case of CIE L*a*b* color model were much better. Due to pressing of finger against surface, the L* value (lightness) is increased. The chromatic value a*, which represents an axis from green to magenta, is significantly decreased and b* chromatic value, which represents an axis from blue to yellow, is increased.

The results of RGB color model are more definite and proper. The biggest difference can be seen always between *G* components. The other differences are lower as follows [28]:

(1) $$\begin{array}{c} \left(G_{2}-G_{1}\right)\;>\;\left(B_{2}-B_{1}\right)\;>\;\left(R_{2}-R_{1}\right), \end{array}$$

where *x*_2_ is average value of *X* in center of image of pressed finger and *x*_1_ is identical calculation for image of nonpressed finger, where *X* is particular component in RGB color model.

**Figure 6:**
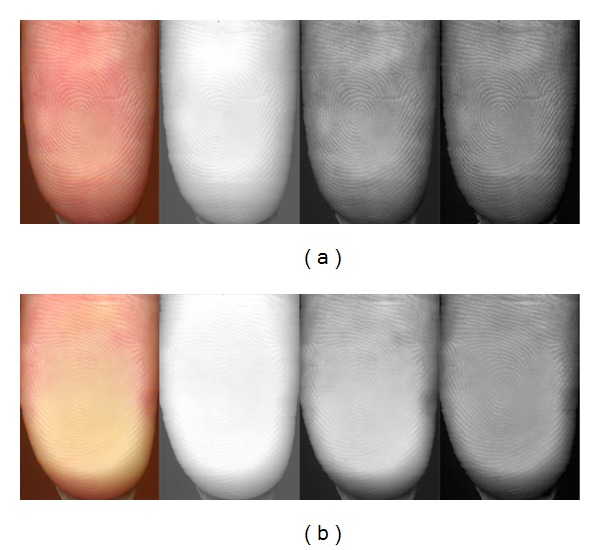
Comparison of nonpressed finger (a) and pressed finger (b). In the first column (from the left), there is the finger in all RGB colors, in the second one, there is only the *R*-channel, the *G*-channel is in the third, and the *B*-channel is in the fourth column. The difference between average *R* values is 11, *G* 42 and *B* 20 [28].

The optical comparison between nonpressed and pressed finger for full RGB image and also decomposed individual components can be found in Figure 6.

The width of papillary lines (and its change) could be detected in various ways. Above all, it will be necessary to choose an appropriate edge detector—a lot of edge detection methods are suitable for this purpose, for example, Sobel filter, Gabor filter, or Canny edge detector. The choice of appropriate method will be highly dependent on the used illumination source(s) mostly considering the angle of light. Moreover, the structure of used pipeline will be important, for example, use of appropriate image preprocessing/postprocessing techniques.

The successful liveness detection mechanism should meet a lot of requirements. According to the described biological principle of both tested characteristics of live human finger, the requirement for universality and permanence should be met. There is no expected problem according to the acceptability requirement. Nevertheless, in [28], these assumptions are checked during select tests by choosing of volunteers of different age, gender, and race and by tests of larger group of volunteers. The requirement for collectability was tested (and met). The requirement for concurrent measuring of the same area without interaction is met in the basis of the method proposal. The liveness detection measurements do not require any special illumination or other interfering hardware, so it is possible to run these measurements simultaneously with the capturing of fingerprint by common optical fingerprint sensor without any risk of negative interaction.

Regarding the requirement for security, it is necessary to ask for resistibility against the known methods of sensor spoofing. There is a lot of possible ways how to create an artificial finger of appropriate color, but there is no skin-color material, which will be able to change the color in the same way as the pressed finger.

The possible way how to pretend the color change is to exchange two fake fingers, each of a different color or to use two inks and to soak the stamp in the second ink during the capturing process. In [28], the exchange of two samples was tested using the Nikon camera (30 fps) and the speed of exchange was only 0.07 seconds. Nevertheless, this situation cannot spoof the proposed liveness detection unit, if the continuous monitoring of the color change will be implemented and the camera with high frame rate will be used.

Forgery of change of papillary lines width is also a nontrivial task. The elasticity of materials for fake finger creation has not been so widely tested. Thus, it is not possible to exclude the eventuality that some of the commonly used materials can have similar properties to the live human skin.

Generally, the common materials usable for fake finger creation to imitate the elasticity of the live human skin can be divided into three groups [28]: *pressure resistant materials* (e.g., sheet of rubber from a common office stamp), *ordinary materials* (e.g., gelatin or latex are often used), and *soft* (easily deformable) *materials*. In case of pressure resistant materials, the change of papillary line width should not be visible. It seems logical to use soft/easily deformable materials and to forge the change of papillary line width by controlling the pressing force. However, such fake fingers are often not able to forge the reverse change (decrease of the pressure and lifting of finger from the sensor surface) due to the slow or even nonexisting memory effect of material. Nevertheless, it is necessary to test various materials during the tests of this approach.

Another possible approach about how to imitate the change of width and color of papillary lines could be the use of thin semitransparent fake finger. Nevertheless, the creation and use of such fake finger could be very difficult (or even impossible), because there are two opposing requirements for the level of transparency. These fake fingers have to be transparent enough to allow to clearly see the color change, and nontransparent enough to allow to clearly see the papillary lines on the fake finger surface noninterfering with the papillary lines from the live finger behind [28]. Moreover, it is necessary to take into account that if the material is not as hard as glass, the finger has to be pressed significantly stronger to achieve the same color change, which influences the change of width of papillary lines on the fake finger surface. Another possible complication for the attacker could be the fact that a lot of commonly used transparent (or semitransparent) materials often contain significant amount of bubbles.

One of very often discussed ways how to spoof fingerprint sensor is the use of dead finger [28]. The capturing of the dead/removed finger may be difficult. It is also known that the color of human skin is conditioned by the circulation of the blood and that the skin due to the lack of blood circulation turns pale/grayish (pallor mortis). According [30], the paleness of skin develops rapidly and it can be easily optically distinguished from the common live skin color. The following postmortem change of skin color is turning dark purple (livor mortis) [30]. This change is caused by gravity and thus it is present only in the lower part of the body. During the first few hours after death, the dark purple parts of skin can turn whitish after applying pressure, but later, this effect is not observable.

There are many physical, chemical, and bacterial decay changes setting after death [27, 31, 32]. Color of the body is ultimately determined by the degree of oxyhemoglobin in the blood present at the time of death. With decreasing oxygen in the blood, the coloration of the skin is changing in the most cases from pink to pale and purplish blue—*pallor* and *livor mortis*. The following color changes are caused by degradation of hemoglobin. Decomposition of soft tissues is coming immediately. It is a process of endogenous autolysis (process of self-digestion by enzymes) and putrefaction (caused by bacterial flora owing to the fact that the body no longer has a functional immune system). Decomposition is progressing with breakdown of blood vessels and extravasation of red blood cells into the subcutaneous and adipose tissues. Soft tissues including skin are going to be disintegrated. Epidermal vesicle formation and skin slippage occur as the epidermis separates from the underlying dermis. Nevertheless, the epidermis commonly retains enough ridge detail to allow fingerprints to be obtained. It assists in the identification of the decedent but also could potentially make impossible identifying a genuine user of biometric systems.

According to the above described color changes of dead skin, this liveness detection approach could be capable to identify the dead finger as a fake finger. Generally speaking, the elasticity could be a little bit weaker than the color change, but coupled together they could create very strong barrier for the possible impostor. The proposed approach could also deal with the capturing of dry, wet, or bended skin, which can be an advantage in comparison with other approaches. Another advantage of this approach is that this method needs not wait until some physiological process (e.g., perspiration or several heartbeats) takes place. When using the hardware with appropriate parameters, the speed of the whole system is limited only by the quality of algorithm implementation. On the other hand, there is also a disadvantage. The proposed approach can have a problem with a high percentage of skin contaminated by colored material (e.g., ink, chalk, or some chemical substances), so the possibilities of deployment of this sensor could be slightly limited. On the other hand, a lot of sensors on the market have a similar problem.

**Figure 7:**
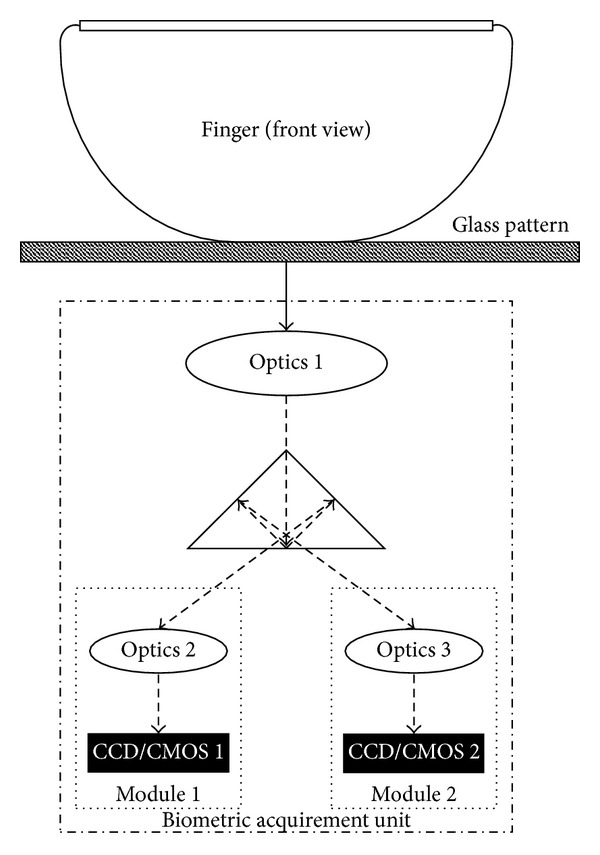
Schema of the proposed sensor.

According to the previously described requirements and the software principle of new method, we proposed the hardware schema of the possible liveness detection unit (This method was registered as the Czech utility model no. 19364 by the Czech Industrial Property Office in 2009.), see Figure 7. This unit can be integrated into an optical fingerprint sensor or it can be used as a sensor with the liveness detection ability (after a few necessary adjustments). In comparison with other partially similar approaches, the proposed liveness detection unit does not need any specific illumination sources, and the common white LED diodes or other ordinary light sources in various locations are sufficient.

The whole unit consists of two camera modules, prism, optics, and glass plate; see Figure 7. The first camera (camera module) will be used for detection of papillary lines width. It is necessary to use the camera with good quality optics to achieve the sufficient magnification of papillary lines, but the camera can have lower image framerate and it can use gray-scale image/video stream. The second camera has to follow the process of color change, so it has to produce a video stream with color images (lower resolution is possible). Nevertheless, this camera will be also used for detection of possible attacks (e.g., by exchanging two different artificial fingers). Because this kind of attack can be done quickly, it is necessary to have the camera with high image framerate (30 fps or better).

It is possible to use only one camera module, but in such case, the “united module” would have to meet all requirements for both separate camera modules. Such solution is currently more expensive and the possibilities of miniaturization are limited as well.

For the purposes of testing of this approach, a new optical bench was created. The bench consists of body, camera mounting module, camera (or other capturing device or other sensor generally), special fingerprint module, and mounting module. This optical bench is designed as multifunctional, so both mounting modules allow to set an arbitrary position (in the corresponding axis) and also to mount different sensors/fingerprint modules, so the whole unit can be used for testing of different configurations and even different ideas (not even for the liveness detection purposes).

A special fingerprint module was developed for the purposes of testing of this approach. This module is intentionally robust, because during preliminary tests, volunteers often feared that they could destroy the facility by pressing too hard. For higher user friendliness, the module has an entrance for a finger from both sides.

### 2.3. Measurement of Optical Changes in the Finger Based on Illumination with Various Wavelengths

A liveness detection method described in the Section 2.2 has a significant disadvantage—it requires a contact between finger and the fingerprint scanner glass. Therefore, its usage is practically limited to touch-based optical sensors. Other types of fingerprint scanners, for example, capacitive scanners, pressure based scanners, e-field scanners, and so forth, cannot use a contact based method because their surface containing capacitors, electro-conductive layers, or small electric field measuring antennas do not allow the presence of glass. Nowadays, also the touchless fingerprint scanners including those based on optical sensing technology are widely used. Contact-based liveness detection methods cannot be used for these sensors from obvious reasons.

Fortunately, a *contactless liveness detection method* compatible with all kinds of fingerprint sensors has been developed [33]. It is based on light illumination with various wavelengths and optical changes measurement. The source of light and the cameras do not need to be placed directly under the scanned finger where usually the fingerprint scanner is placed. For example, the source of light can be placed on one side of scanning part and the camera on the other side. This is the most convenient method for all touchless scanners.

This method is based on the following principle. A light of specific wavelength emitted from a light source mounted into fingerprint scanner illuminates a scanned fingertip. In this way, the part of emitted electromagnetic radiation encountering some object is absorbed by the material, part of this light is reflected back, and part of the light goes through the object. The main point of our interest is a light reflected from the fingertip surface and from the nearby sublayers. For the liveness detection purposes, an amount of the reflected part of light is measured. A live finger due to its physical attributes (blood oxygenation, temperature, etc.) has different spectral properties than the dead or fake finger. Also the amount of reflected light of a specific wavelength should differentiate the live finger from the other ones.

**Figure 8:**
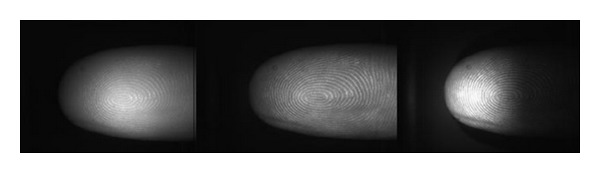
Finger illuminated by 700 nm red light, 550 nm green light, and 470 nm blue light.

For the experiments, the following light wavelengths were selected: 700 nm (red), 550 nm (green), and 470 nm (blue). The illuminated fingerprints were captured by a high-resolution camera and saved as a 1,280 × 1,024 pixel grayscale images. The example of scanned fingerprints can be seen in Figure 8.

**Figure 9:**
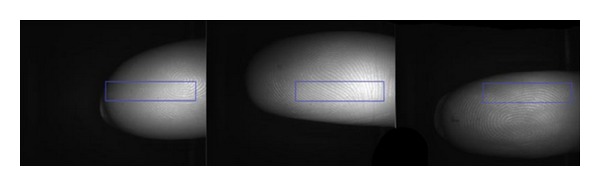
Rectangle for feature extraction.

Before determination of suitable features, an image preprocessing has to be performed. The fingerprint image background has to be excluded from further processing because the black background pixels can strongly influence the global features extracted from the image used for liveness detection. After scanning a large amount of fingerprints, we were able to determine a fixed region of interest (ROI) in the image invariant to the finger position where no background is present. The example of different finger positions with the determined ROI highlighted can be seen in Figure 9. This ROI is used afterwards for the feature detection. From the determined rectangle, it is possible to extract local and the global features. 

**Figure 10:**
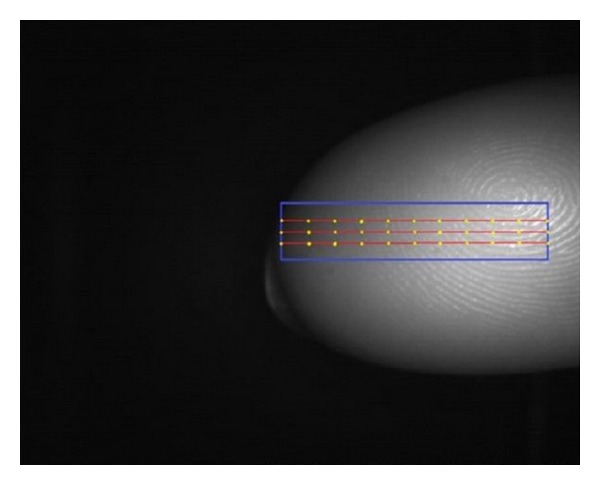
Local feature extraction points.

As a local feature, for example, a pixel intensity arithmetic mean or the pixel intensity standard deviation from only some selected pixels could be considered. During our experiments, we determined three line segments inside the rectangle, each segment containing 11 pixels of interest from which the local features were extracted. The visualization of line segment position and pixels of interest position can be seen in Figure 10.

As a global feature extracted from the whole rectangle, the following statistics indicators were considered: pixel intensity arithmetic mean, pixel intensity standard deviation, pixel intensity median, histogram mean, histogram standard deviation, and the histogram median.

**Figure 11:**
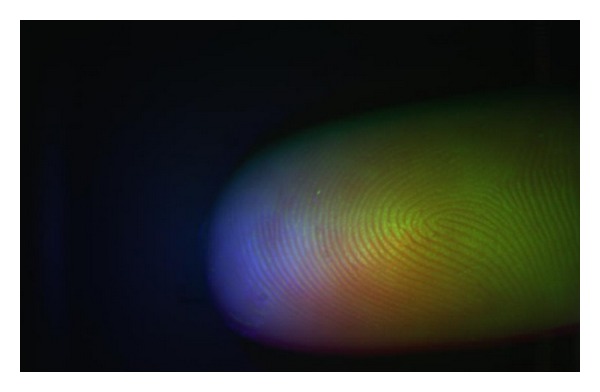
Color image obtained by merging the original images (RGB color model).

Having captured the three grayscale images of finger illuminated by three different wavelengths, it is possible to merge these three one-channel images into one three-channel color image. Some significant features could be extracted. We used the analysis of 3-channel histogram. During our experiments, we considered several color models. By simply merging the input images, we got the RGB representation of fingerprint. Image in RGB gained by merging the images from Figure 8 can be seen in Figure 11.

**Figure 12:**
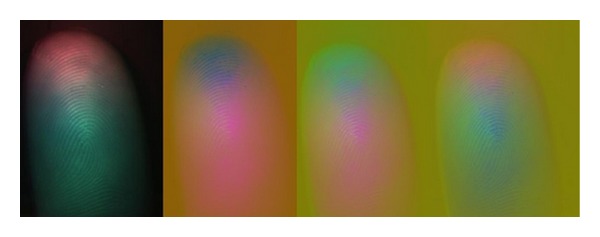
Color image converted into XYZ, LUV, LAB, and YCbCr color model.

In image processing and computer vision areas, the RGB color model is not always the best option. By converting the color image into some other color models, the precision of live-finger decision could be significantly increased. The following color models were considered: XYZ color model (1931 CIE), LUV color model (CIE 1976), LAB color model (CIE 1976), and the YCbCr color model.  Figure 12 shows the results of the color model conversions. The previously mentioned features can be extracted from any color image.

All the extracted features from a specific color model are concatenated to one feature vector. Feature vectors from various real and artificial fingers are used as the training data for the selected machine-learning method. For our purposes, we use artificial neural networks and random forests [13, 34].

We evaluated performance of each proposed algorithm by 8 cross-validation runs on the database containing 150 fingerprints [33]. The best result was achieved by combination of Luv and YCbCr model. The FRR was less than 2% while FAR was 10%. These results can be further improved by applying more LED with different wavelengths and more advanced feature extractors.

### 2.4. Skin Diseases and Their Influence on Liveness Detection

Skin diseases could represent a serious problem in the process of liveness detection. In a general medicine, about 20%–25% of patients suffer from some skin disorder. In this paper, we discuss the diseases localized on palmar side of hands including fingertips. Some diagnoses are typical for this localization; others affect skin surface generally comprehending palms and fingers. When discussing whether the fingerprint recognition technology is a perfect solution capable to resolve all security problems, we should always keep in mind those potential users who suffer from some skin diseases.

The border of *epidermis* and *dermis* (dermoepidermal junction) forms the base of papillary lines. In most cases of dermatological disorders, we find a lot of changes in the ultrastructure of the skin, including epidermis and dermis. There is often inflammation, atrophy or hypertrophy, fibrotisation, and many other changes visible in the microscope. These differences result in changes of color (optical characteristics), changes of dermal vessels and capillaries (blood perfusion), and changes of elasticity and thickness of the skin (optical characteristics after pressure change).

Some examples of skin diseases potentially making impossible the liveness detection are the following ones. The description is made from a medical point of view.

**Figure 13:**
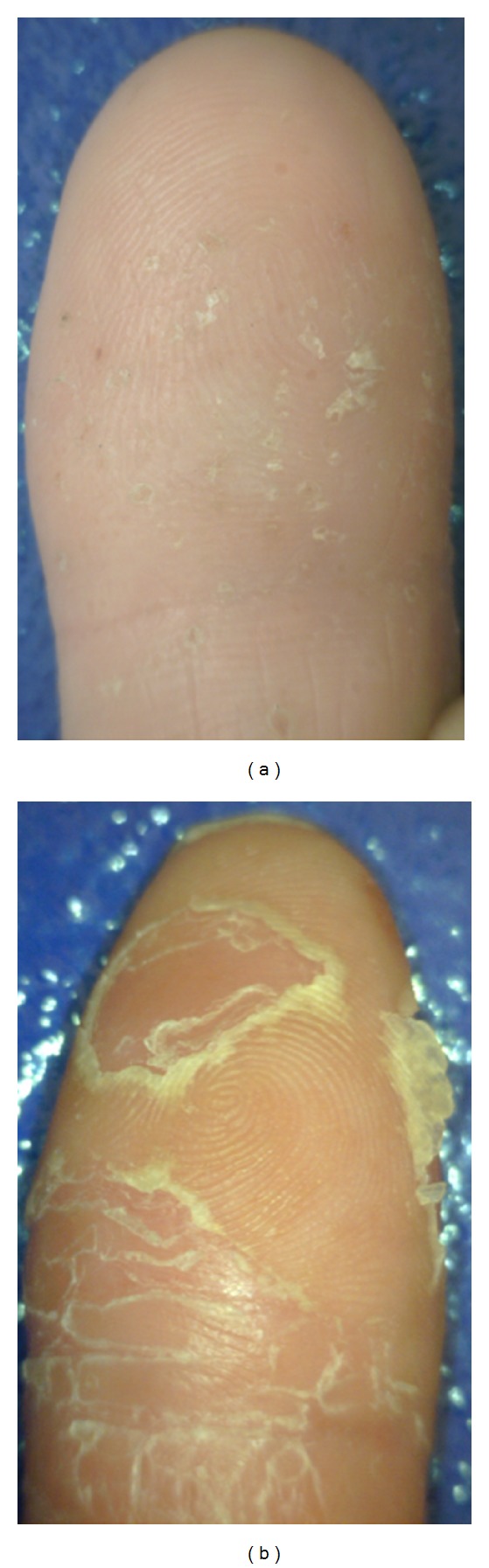
(a) Fingertip eczema; (b) psoriasis.

*Hand and fingertip eczema *[35, 36] (see Figure 13(a)) is an inflammatory noninfectious long-lasting disease with relapsing course. It is one of the most common problems encountered by the dermatologist. Hand dermatitis causes discomfort and embarrassment and, because of its locations, interferes significantly with normal daily activities. Hand dermatitis is common in industrial occupations. The prevalence of hand eczema was approximately 5.4% and was twice as common in females as in males. The most common type of hand eczema was irritant contact dermatitis (35%), followed by atopic eczema (22%) and allergic contact dermatitis (19%). The most common contact allergies were to nickel, cobalt, fragrance mix, balsam of Peru, and colophony. Hand eczema was more common among people reporting occupational exposure. The most harmful exposure was to chemicals, water and detergents, dust, and dry dirt.

**Figure 14:**
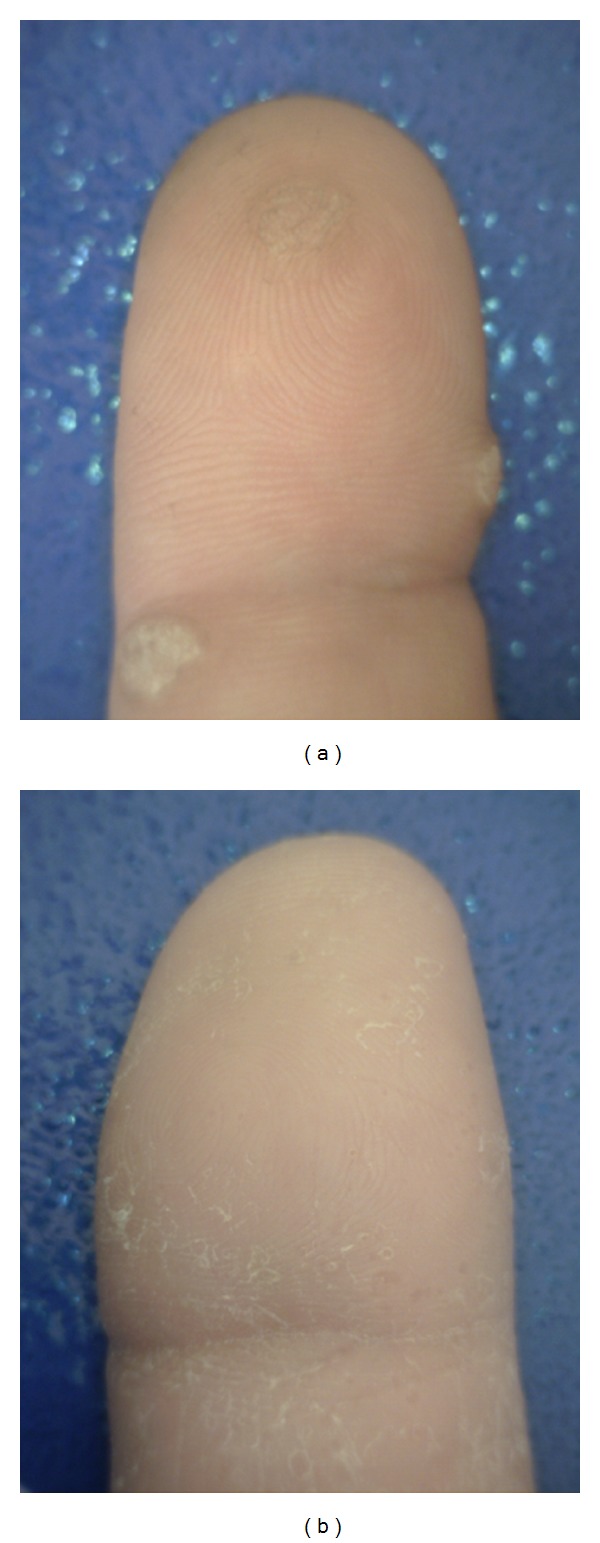
(a) Verruca vulgaris; (b) dyshidrosis.

*Warts* (*verruca vulgaris*) [29, 37] (see Figure 14(a)) are benign epidermal neoplasms that are caused by human papilloma viruses (HPVs). Warts commonly appear at sites of trauma, on the hand, in periungual regions. HPVs induce hyperplasia and hyperkeratosis.

*Psoriasis *[27, 38] (see Figure 13(b)) is characterized by scaly papules and plaques. It occurs in 1% to 3% of the population. The disease is transmitted genetically; environmental factors are needed to precipitate the disease. Psoriasis of the palms and fingertips is characterized by red plaques with thick grey scale and may be indistinguishable from chronic eczema.

*Pompholyx* (*dyshidrosis*) [39] (see Figure 13(b)) is a distinctive reaction pattern of unknown etiology presenting as symmetric vesicular hand and foot dermatitis. Itching precedes the appearance of vesicles on the palms and sides of the fingers. The skin may be red and wet.

*Tinea of the palm* [35] is dry, diffuse, keratotic form of tinea. The dry keratotic form may be asymptomatic and the patient may be unaware of the infection, attributing the dry, thick, scaly surface to hard physical labor. It is frequently seen in association with tinea pedis whose prevalence is 10 to 30%.

*Pyoderma* [29] is a sign of bacterial infection of the skin. It is caused by *Staphylococcus aureus* and *Streptococcus pyogenes*. Some people are more susceptible to these diseases (such as diabetics, alcoholics, etc.).

*Systemic sclerosis* [27] is a chronic autoimmune disease characterized by sclerosis of the skin or other organs. Emergence of acrosclerosis is decisive for fingerprinting. Initially the skin is infused with edema mainly affecting hands. With the progressive edema stiff skin appears and necrosis of fingers may form. For more than 90% of patients is typical Raynaud's phenomenon (see below). The typical patient is a woman over 50 years of age.

*Raynaud's phenomenon *[35] represents an episodic vasoconstriction of the digital arteries and arterioles that is precipitated by cold and stress. There are three stages during a single episode: pallor (white), cyanosis (blue), and hyperemia (red). 

*Erythema multiforme* [29] is quite common skin disorder with multifactorial cause. The most common triggering agents are infections (in the first place herpes virus) and drugs. Both forms are characterized by erythematous target-shaped lesions with a center with hemorrhage, blistering, necrosis, or crust.

*Epidermolysis bullosa *[27] is a term given to groups of genetic diseases in which minor trauma causes noninflammatory blistering (mechanobullosus diseases). Repetitive trauma may lead to a mitten-like deformity with digits encased in an epidermal “cocoon.” These diseases are classified as scarring and nonscarring and histologically by the level of blister formation.

*Dermatitis artefacta *[35, 40, 41] are changes of skin due to the manipulation by patient. Patients often have psychosomatic, psychiatric, or drug abuse problems.

*Cutaneous adverse drug reactions* [35] occur in many forms and can mimic virtually any dermatosis and they occur in 2%-3% of hospitalized patients. Antibiotics, sulfonamides, some nonsteroidal antiphlogistics, and anticonvulsants are most often applied in the etiology.

## 3. Conclusion

This paper introduces fake finger use and liveness detection in general at the beginning. The ways of possible attacks to fingerprint-based biometric systems are described as well. The main part of this paper is devoted to three new methods of liveness detection suitable for fingerprint recognition systems. The first method is based on pulse detection (heart activity), which is measured by a laser distance measurement unit (triangulation principle)—the pulse curvature in the acquired data is comparable with ECG signal. This first method is patented by us—see [26]. The second approach is based on detection of color change of the fingertip skin and thickness of papillary lines after the application of higher pressure to a glass platen. The color change could be distinguished in various color models, for example, RGB. The second method is registered by us as a national utility model by the Czech Industrial Property Office under the no. 19364 (http://isdv.upv.cz/portal/pls/portal/portlets.pts.det?xprim=1064464) (2009). The last method is based on reaction of skin on the fingertip to illumination with various wavelengths—this principle is not fully new, and the company Lumidigm Inc. has some patents in this area. Anyway we realized new experiments and these are published in this paper for the first time. At the end of this paper, the possible influence of skin diseases on liveness detection is discussed, because the skin diseases could really influence the liveness detection so that the live finger could be (due to any dermatologic problem) classified as a nonliving finger or artificial fake finger. The most influencing skin diseases are described in the last section.
